# Measurement of sustainable higher education development: Evidence from China

**DOI:** 10.1371/journal.pone.0233747

**Published:** 2020-06-01

**Authors:** Yuqing Geng, Nan Zhao

**Affiliations:** School of Business, Shanghai Dianji University, Shanghai, China; Institute for Advanced Sustainability Studies, GERMANY

## Abstract

This paper constructs the 6E evaluation index system, a comprehensive index including the dimensions of economy, effectiveness, efficiency, equity, earnings and equality, to measure the sustainable higher education development of the 31 provincial regions of China by utilizing the information entropy weight-TOPSIS method. This paper then makes a spatial and temporal analysis of the coupling coordination relationship among the dimensions of sustainable higher education development by using the coupling coordination model. In addition, this paper proposes specific and applicable countermeasures for sustainable higher education development. The results show that the comprehensive degrees of sustainable higher education development in most regions are not high, and the coastal regions and the Central-south China regions have higher grades; in addition, for most regions, the coupling coordination degrees mainly remain stable, with mild growth in the respective classifications, and the gap between the west and other regions is declining. The improved method is applicable to measure the sustainable development of higher education and to propose detailed and appropriate suggestions for further development.

## Introduction

In the early 1980s, Romer [1] proposed a new theory of economic growth, stressing that higher education is important to improve the regional economy and human capital. This theory is well supported by the proposition of sustainable development in education [2], the implementation of higher education development strategies in many countries [3], and the success of countries’ economic growth and talent training [4][5]. In addition, higher education institutions can help foster human sustainability [6]; thus, developing higher education institutions with a sustainable perspective is a key focus. Before the 1990s, the concepts of University 1.0, 2.0 and 3.0, highlighting the aims of universities, were proposed. University 1.0 was primarily concerned with the development of educational institutions themselves; University 2.0 mainly focused on the coordination of teaching and research of universities [7], and University 3.0 added a new aspect: the contribution of knowledge to society and economics [8]. These three versions of the aims have become the basis of higher education institutions’ competitiveness and have received much attention from stakeholders. With the renown and common sense of sustainability, University 4.0 is hereby proposed, illustrating that the development of higher education institutions should consider the sustainability of both the institutions themselves and society [9].

The United Nations (UN) proposes the connotation of sustainable development in education, and many countries have initiated programs to accelerate sustainable higher education development (SHED) [2]; however, higher education still performs weakly with respect to sustainability, and regional imbalances of SHED still exist [10,11]. In addition, particular factors exist in different regions to hinder the advancement of SHED, and the coordination mechanism among the factors affecting SHED is not clear [12]. Therefore, it is important to clarify the coordination mechanism among the factors of SHED theoretically and empirically and to explore efficient approaches to measure SHED.

This paper aims to construct an evaluation framework with an index system that can evaluate SHED, to analyze the status of SHED, to measure the coupling coordination relationship of the dimensions within SHED and to propose specific countermeasures to enhance SHED through coordination. The paper is organized as follows. Section 2 is the literature review; section 3 is the introduction of the study area and data sources; section 4 is the construction of the SHED evaluation framework and index system and the introduction to the evaluation process; section 5 is the discussion of the results; section 6 gives specific countermeasures of SHED; and in the final part, section 7, conclusions and future prospects are given.

## Literature review

The sustainability of higher education refers to coordinated development involving the aspects of environment, economics, culture, gender equality, community responsibility, etc. [13–16]; the long-term balance among them should be maintained [17]. The concept of SHED is comprehensive, including the interaction of higher education with the surrounding environment, economic growth, societal equity, equality, quality enhancement, etc. Therefore, once the definition of SHED can be identified, we can determine indicators to measure SHED and its coordination status [18].

Current studies are trying to determine the goals and aims of SHED and illustrate the correlation between higher education and other aspects (such as environment, society, culture, economy). To find appropriate influencing factors to measure SHED, studies seek approaches to measure SHED [19]. In detail, the UN proposes the following dimensions of sustainability in education: inclusiveness, gender equality, qualified education, lifelong education, and social interactions [20,21]. Berchem proposes that competitive higher education depends on scientific research, economic contributions, and social and cultural interactions [22]. By building research labs or scientific centers, universities construct relationships with industry; thus, higher education institutions develop in view of economic output [23–25]. Furthermore, one of the aims of higher education is to cultivate competitive students in the job market [26,27]; thus, the cultivation of students with competitive knowledge is considered a factor of SHED [28,29]. In addition, the UN proposes that international cooperation is one of the goals of sustainable education [2], meaning that internationalization is one of the factors that describes SHED. In addition, higher education institutions are suggested to admit both domestic and international students to increase their potential competitiveness in the global labor market [30] and to provide students with lifelong higher education opportunities to enhance the competency of the higher education institutions [31]. The generalization and massification of higher education are also mentioned as the goals of SHED, and these factors influence university development [32]. The generalization and massification of higher education are affected not only by population growth but also by changes in the population and industry structures [33,34]; thus, the number of students and the relation with industry can be applied to evaluate SHED. Furthermore, scholars discuss the development of higher education from the perspective of the benefits of persons, demonstrating that human development and welfare are key aspects for evaluating SHED [35,36]. Although there are studies on the goals of SHED, there is no broad consensus regarding how to comprehensively measure SHED with its components’ interactions.

Efforts have been made to evaluate SHED from different perspectives, but the approaches to measure SHED are still one of the main issues to be discussed [37]. Scholars have conducted a variety of studies on this topic via qualitative or quantitative approaches. Qualitative research mainly focuses on the origin and strategies of SHED [38,39], while quantitative research mainly evaluates the sustainable development ability and competitiveness of higher education [31,40], analyzes related factors affecting SHED [41], explores the coordination mechanism between higher education and other factors [42], etc. However, previous studies are mainly based on specific cities or universities, and the spatial-temporal interprovincial comparisons of SHED components are relatively inadequate. In addition, there are debates in the evaluation process; weighting is a key step in the SHED measurement procedure and can be divided into two categories: the subjective method and the objective method. In the subjective method, professionals determine the weighting coefficient of indicators by marking scores. The typical subjective methods are the Delphi method [43] and analytic hierarchy process (AHP) [44]. The objective approach is used to determine the weight of indicators based on the factual data to avoid the deviation of factors and personal bias. The typical methods include cluster analysis [31], gray correlation analysis (GCA) [45], principal component analysis (PCA) [46], information entropy weight analysis (IEW) [47], and the technique for order preference by similarity to an ideal solution (TOPSIS) [47]. Previous research has proven the deficiencies of the methods above. For instance, subjective weighting methods, such as the AHP and Delphi methods, are proven to have poor objectivity and preciseness because of personal preferences and bias; PCA is likely to lose information in the extraction of main components that are related to the indicator numbers; GCA and cluster analysis are suitable if they are used jointly with other methods, such as PCA and AHP, but they perform poorly when used independently in the index weight determination [48]. Current studies taking measurements in the field of higher education mainly use subjective approaches that neglect the importance of data, so the results are not always convincing. In contrast, the combination of IEW and TOPSIS is an improved approach in which the weights of the indices are determined according to the combined weighting method. The ranking of the objects is decided by the ranking that approximates the ideal solution [49]. IEW-TOPSIS can reflect the relative importance of the indicators using data, making it highly applicable and convincing to the measure SHED. Applying IEW-TOPSIS to the measurement of SHED can achieve the intended purpose and represent advantages over other existing approaches.

In conclusion, great efforts have been made in defining and evaluating SHED, but currently, there are no widely accepted and applicable evaluation metrics to measure SHED, so the attempt to measure the performance of SHED and the interaction mechanisms among its dimensions is difficult. In addition, current studies mainly focus on SHED from microscopic perspectives, such as at the university or individual regional level, ignoring interregional comparisons, although spatial-temporal comparisons from a macroscopic perspective are helpful for us to better understand SHED completely and comprehensively. In addition, the former methods used to evaluate SHED are insufficient, and an improved method is needed to better measure SHED. Therefore, it is necessary to construct an accepted and applicable evaluation framework to measure SHED and use it for temporal and spatial evaluation and comparison with an improved objective approach.

## Study areas and data sources

### Study areas

**Fig 1:**
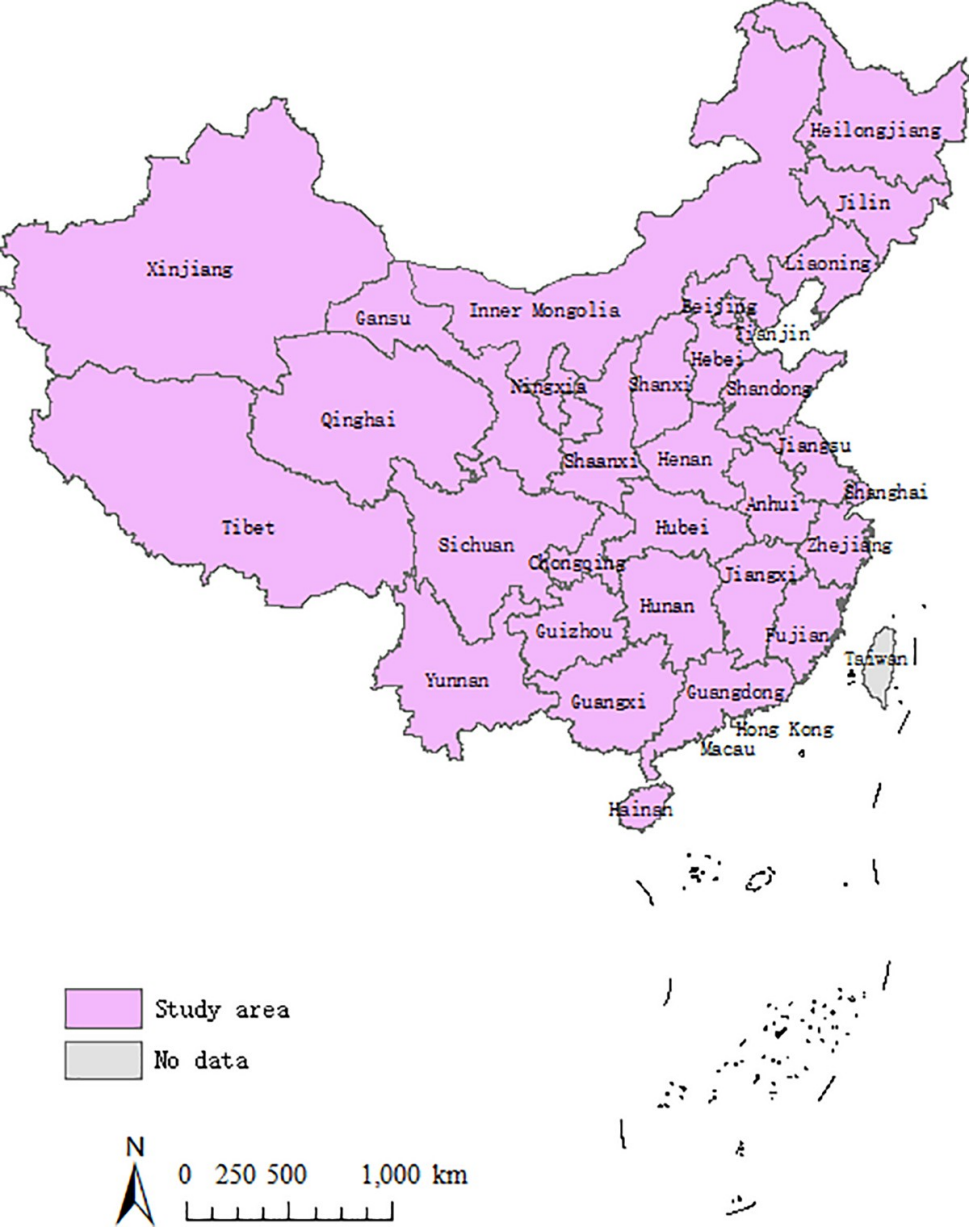
Study area.

As one of the largest countries globally, China enjoys approximately 9600 square kilometers and consists of 34 provincial administrative units, including 23 provinces, 4 municipalities, 5 ethic autonomous regions, and 2 special administrative regions. China has recently witnessed rapid development in sustainability in various fields, including higher education. Its higher education is playing a competitive role: according to the statistics from the Educational Statistics Yearbook of China, in 2018, there were 7.53 million undergraduates and 0.60 million postgraduates graduating from 2663 higher education institutions, 3,889 billion yuan invested in education, 1.31 million scientific papers issued, 45,591 science and technology publications, and 278 thousand patents applications. However, a regional imbalance of SHED and conflicts of resource allocation have accompanied the recent rapid growth of higher education in China. Therefore, taking the provincial units of China as research objects and measuring the development status and the coupling coordination of SHED are of importance to enhance the regional balance and accelerate the sustainability of higher education in different regions. In this study, 31 provincial units are selected as research objects, while 3 units (Hong Kong, Macau and Taiwan) are excluded due to significant differences. The 31 objects are Yunnan, Hebei, Shandong, Henan, Guizhou, Shanxi, Heilongjiang, Jiangsu, Liaoning, Jilin, Fujian, Jiangxi, Hubei, Shaanxi, Guangdong, Gansu, Hunan, Anhui, Sichuan, Hainan, Zhejiang, Qinghai (22 provinces), Beijing, Tianjin, Shanghai, Chongqing (4 municipalities), Inner Mongolia, Tibet, Xinjiang, Ningxia, and Guangxi (5 ethic autonomous regions). (Fig 1)

The choice of the provincial regions in China as the study case can make the research representative. First, China is the largest developing country in the world and has experienced rapid development and large regional differences in recent decades, and research on SHED in China can promote understanding of the development of higher education in emerging countries. Second, the current research mainly focuses on specific regions and lacks spatial and temporal comparisons among regions in measuring SHED, and this paper can fill this gap by comparing the provincial differences among the 31 regions in China.

### Data sources

The data obtained are from the China Statistical Yearbook (2013–2017), Educational Statistics Yearbook of China (2013–2017), China Statistical Yearbook on Sciences and Technology (2013–2017), and China Torch Statistical Yearbook (2013–2017). Data of certain indicators are calculated and converted through original materials with formulas.

## Study methods

### Construction of the evaluation framework

**Fig 2:**
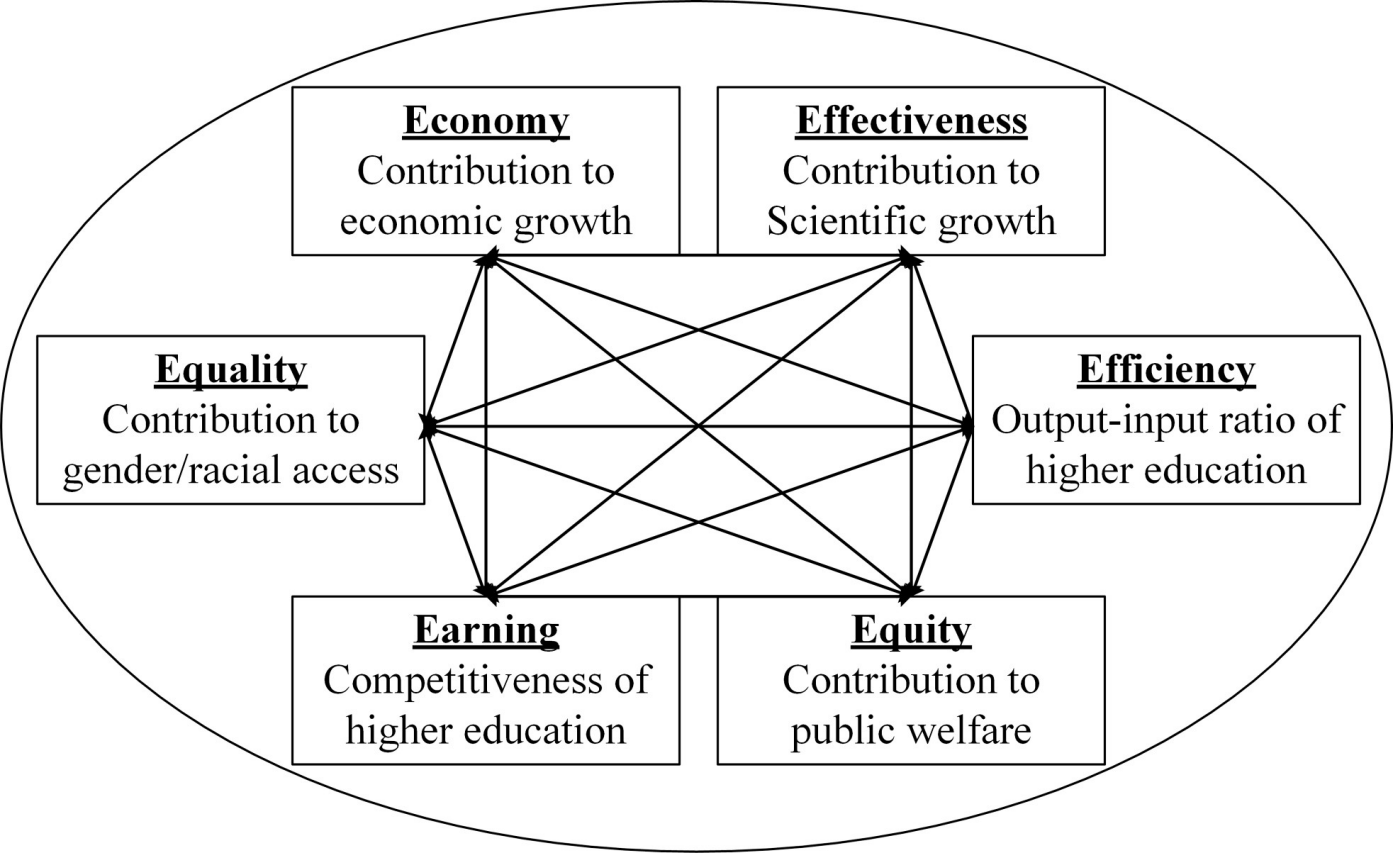
6E evaluation framework.

From the sustainability perspective, the evaluation framework of SHED should be more comprehensive than other frameworks evaluating business or industries because we need to consider the sustainable coordination mechanism among the dimensions of SHED. According to the review of the above literature, the evaluation framework of SHED should focus on equity and equality, which are the requirements of sustainable education proposed by the UN. In addition, economy, effectiveness, efficiency, and earnings should be introduced into the framework of SHED. Economy refers to the sustainable contribution of higher education to the economic output; effectiveness refers to the sustainable contribution of the research output to organizational targets; efficiency refers to the ratio of output and input and evaluates whether the higher education of a region develops efficiently with sustainability; equity refers to the sustainable contribution to the interaction with the society, including the education service provided; earnings refer to the annual performance, which illustrates the quality enhancement of higher education; and equality refers to the contribution of gender and racial equality to higher education development. These 6 dimensions can be summarized as 6E according to the initial letter of the words and can comprehensively illustrate the coordinated performances of SHED. The 6E framework is a new and advanced one compared with the former frameworks, as it consists of economy, effectiveness, efficiency, equity, equality and earnings from the sustainability perspective and addresses the coordination mechanism of the SHED dimensions (Fig 2).

### Construction of the evaluation index system

According to the 6E evaluation framework, indicators are screened and selected to measure SHED (Table 1). The selection principles are as follows. First, the indicators should be representative in illustrating SHED; second, the indicators should be widely accepted and cited and easy for the public to understand; third, the indicators should be applicable after eliminating multicollinearity; and fourth, the data of the indicators should be accessible [50].

**Table 1:** Evaluation index system of SHED.

System	Dimension	Indicator
Sustainable higher education development (A1)	Economy (B1)	Area of incubation space of NUSP (C1)
		Number of new tenants in NUSP (C2)
		Income of the incubation corps. in NUSP (C3)
		Taxes of incubation corps. in NUSP (C4)
	Effectiveness (B2)	Number of scientific papers issued (C5)
		Number of publications on S&T (C6)
		Number of patents (C7)
		Number of R&D projects (C8)
	Efficiency (B3)	Number of students per 100,000 population (C9)
		Student-teacher ratio (C10)
		Funds per student (C11)
		R&D radio of input of funds to expenditures (C12)
		Ratio of doctor's degrees to the number of full-time teachers (C13)
	Equity (B4)	Number of part-time teachers (C14)
		Number of enrolled postgraduate students (C15)
		Number of enrolled undergraduate students (C16)
		Number of students enrolled in self-directed classes (C17)
		Number of students in in-service training (C18)
	Earnings (B5)	Number of HEIs (C19)
		Amount of educational personnel (C20)
		Floor area of school buildings (C21)
		Areas occupied by HEIs (C22)
		Number of books & magazines in libraries (C23)
		Number of PCs (C24)
		Number of classrooms (C25)
		Amount of fixed assets (C26)
	Equality (B6)	Ratio of female postgraduates (C27)
		Ratio of female undergraduates (C28)
		Ratio of female R&D personnel (C29)
		Ratio of female educational personnel (C30)
		Ratio of international graduates (C31)

(1) Economy refers to the contribution of SHED to economic development. The economic contribution of higher education is mainly indirect, so the statistics of National University Science Parks (NUSPs) supported by higher education institutions (HEIs) are selected to evaluate the economic contribution of higher education. In detail, there are 4 indicators: area of incubation space of an NUSP, number of new tenants in the NUSP in that year, income of the incubation corporations in the NUSP, and taxes paid by incubation corporations in the NUSP.

(2) Effectiveness refers to the outcomes and performance of HEIs corresponding to their goals for research and science output. It represents the quality of SHED in science and technology. In this dimension, there are 4 indicators: numbers of scientific papers issued, publications on science & technology, patents, and research & development projects.

(3) Efficiency refers to the input-output ratio of higher education to evaluate SHED efficiency. The main participants and stakeholders, such as faculty, students, fiscal investors, etc., are considered, and the evaluation indices based on the ratio between output and input are selected. There are 5 indicators, namely, the number of students per 100,000 population, student-teacher ratio, funds per number of students, R&D radio of input of funds to expenditures, and ratio of teachers with a PhD to the number of full-time teachers.

(4) Equity refers to both the public participation in higher education and the public welfare from higher education. To achieve the goal of sustainable development, it is important for HEIs to communicate and interact positively with communities and the public; therefore, the interaction with the public is reflected in this dimension. Five indicators are selected to evaluate the participation and benefits of the public. In detail, the indicator “number of part-time teachers” demonstrates the input from the public; the numbers of enrolled postgraduate students, enrolled undergraduate students, students enrolled in self-directed classes, and students in in-service training are the remaining 4 indicators used to evaluate the public welfare from SHED.

(5) Earnings refer to the annual performance of HEIs together with their infrastructures, which directly illustrates SHED from the perspective of quality. Eight indicators are selected in this dimension, including the numbers of HEIs, educational personnel, books & magazines in libraries, PCs, classrooms, fixed assets, floor area of school buildings, areas occupied by HEIs, etc. These indicators comprehensively represent the condition of assets and resources of HEIs in terms of absolute quantity.

(6) Equality refers to the equality of gender and race; in other words, this dimension represents the participation and contribution of women and international interactions to SHED. Gender and racial equality are goals for achieving sustainability in education, so this study adds “equality” to evaluate whether women and international students enjoy equal opportunities in achieving and supporting higher education. Women and international students are the main participants in HEIs either as faculty or as students; therefore, 5 indicators are selected from the student and faculty perspectives: ratios of female postgraduates, female undergraduates, female R&D personnel, female educational personnel, and international graduates. The ratios illustrate the equality of women and international students in SHED.

Compared with former frameworks and indicators in the measurement of SHED, the 6E evaluation framework with the index system presents several advantages. The 6E framework includes 6 dimensions and 31 indicators and is more comprehensive than other alternatives that may ignore certain key dimensions of SHED; in particular, the 6E framework considers the economic contribution of higher education (economy) and emphasizes the contribution of women (equality), which is not common in the previous alternatives and is also more suitable to measure the sustainability of higher education. In addition, the indicators are representative and easy to apply; thus, the index system is likely to be widely accepted and adopted in future research.

### Calculation of the comprehensive degree of SHED

TOPSIS is an effective technique for ranking alternatives by measuring the Euclidean distance. As a multicriteria decision-making approach, TOPSIS proposes that the alternatives being evaluated have the shortest distance from the positive ideal solution (SS) and the longest distance from the negative ideal solution (LS). TOPSIS can be used in many industries and fields. In this paper, IEW and TOPSIS are jointly utilized to achieve the research goals. The detailed processes are as follows.

(1) Calculate the normalized value $x_{ij}^{\prime }$. *i* is the alternative (*i* = 1,2,…,m), *j* is the indicator (*j* = 1,2,…,n), and *x*_*ij*_ is the value in the original matrix.

$$x_{ij}^{\prime }=\frac{x_{ij}}{\sum_{i=1}^{n}x_{ij}}$$ (1)

Here, we define $\underset{1\leq j\leq n}{max}x_{ij}$ and $\underset{1\leq j\leq n}{min}x_{ij}$ as the maximum and the minimum values of indicator *j*.

(2) Calculate the information entropy *IE*.

$${IE}_{j}=-\left(\sum_{i=1}^{m}f_{ij}\;\ln\;fij\right)$$ (2)

where

$$f_{ij}=\frac{1+x_{ij}^{\prime }}{\sum_{i=1}^{m}\left(1+X_{ij}^{\prime }\right)}$$ (3)

(3) Calculate the weight *W*_*j*_.

$$W_{j}=\frac{1-{IE}_{j}}{n-\sum_{j=1}^{n}{IE}_{j}}$$ (4)

(4) Calculate the positive and negative ideal solutions P and N.

$$P=(\underset{1\leq i\leq m}{max}x_{i1},\underset{1\leq i\leq m}{max}x_{i2},\ldots ,\underset{1\leq i\leq m}{max}x_{in})$$ (5)

$$N=(\underset{1\leq i\leq m}{min}x_{i1},\underset{1\leq i\leq m}{min}x_{i2},\ldots ,\underset{1\leq i\leq m}{min}x_{in})$$ (6)

(5) Calculate the separation measure.

$$SS=\sqrt{\sum_{j=1}^{n}w_{j}{\left(x_{ij}-P_{j}\right)}^{2}}$$ (7)

$$LS=\sqrt{\sum_{j=1}^{n}w_{j}{\left(x_{ij}-N_{j}\right)}^{2}}$$ (8)

(6) Calculate the relative closeness *C*, which is named the comprehensive degree of SHED (C-SHED). The larger the value of C is, the better the status of SHED.

$$C=\frac{LS}{LS+SS}$$ (9)

(7) Establish the evaluation grade of the C-SHED. The grade is constructed according to the equalization concept, which guarantees the equality of intervals (Table 2).

**Table 2:** Evaluation grade of C-SHED.

Value	*C* ≥ 0.75	0.50 ≤ *C* < 0.75	0.25 ≤ *C* < 0.50	*C* < 0.25
Grade	Excellent	Good	Fair	Poor

### Calculation of the coupling coordination degree of SHED

SHED in this study is combined with 6 dimensions according to the framework. There are correlations among these dimensions, which restrict or enhance each other and evolve coordinatively. Coupling refers to the notion that the dimensions in SHED interact with each other, and coordination refers to the sustainable relationship among the dimensions when they work together harmoniously [50]; coupling coordination degree is used to measure the interactions of coupling coordination status among the dimensions [51]. Using the coupling coordination model, this study calculates the coupling coordination degree of SHED (D-SHED); the procedures are as follows.

(1) Calculate the coupling degree *CP*, where Q(x), G(y), H(z), K(u), L(u) and M(u) are the C-SHED of the 6 dimensions.

$$CP={\left\{\frac{Q(x)\times G(y)\times H(z)\times K(u)\times L(u)\times M(u)}{{\left(\frac{Q(x)+G(y)+H(z)+K(u)+L(u)+M(u)}{6}\right)}^{6}}\right\}}^{\frac{1}{6}}$$ (10)

(2) Calculate the coordination degree CO. α, β, γ, δ, ε, and ζ are the coefficients. Referring to the existing research [42] and the characteristics of SHED, the dimensions contribute equally to SHED, and therefore, α = 0.17, β = 0.17, γ = 0.17, δ = 0.16, ε = 0.17, and ζ = 0.16.

CO=αQ(x)+βG(y)+γH(z)+δK(u)+εL(u)+ζM(u) (11)

(3) Calculate the coupling coordination degree *DE*.

$$DE=\sqrt{CO\times CP}$$ (12)

Referring to previous research [51], the evaluation classification of *DE* is constructed (Table 3).

**Table 3:** Evaluation classification of D-SHED.

Value of *DE*	Classification
1 ≥ *DE* ≥ 0.8	High coordination
0.8 > *DE* ≥ 0.7	Intermediate coordination
0.7 > *DE* ≥ 0.6	Primary coordination
0.6 > *DE* ≥ 0.5	Reluctant coordination
0.5 > *DE* ≥ 0.4	Approaching imbalance
0.4 > *DE* ≥ 0.3	Slight imbalance
0.3 > *DE* ≥ 0.2	Moderate imbalance
0.2 > *DE* ≥ 0	High imbalance

## Results and discussion

### Analysis of C-SHED

**Fig 3:**
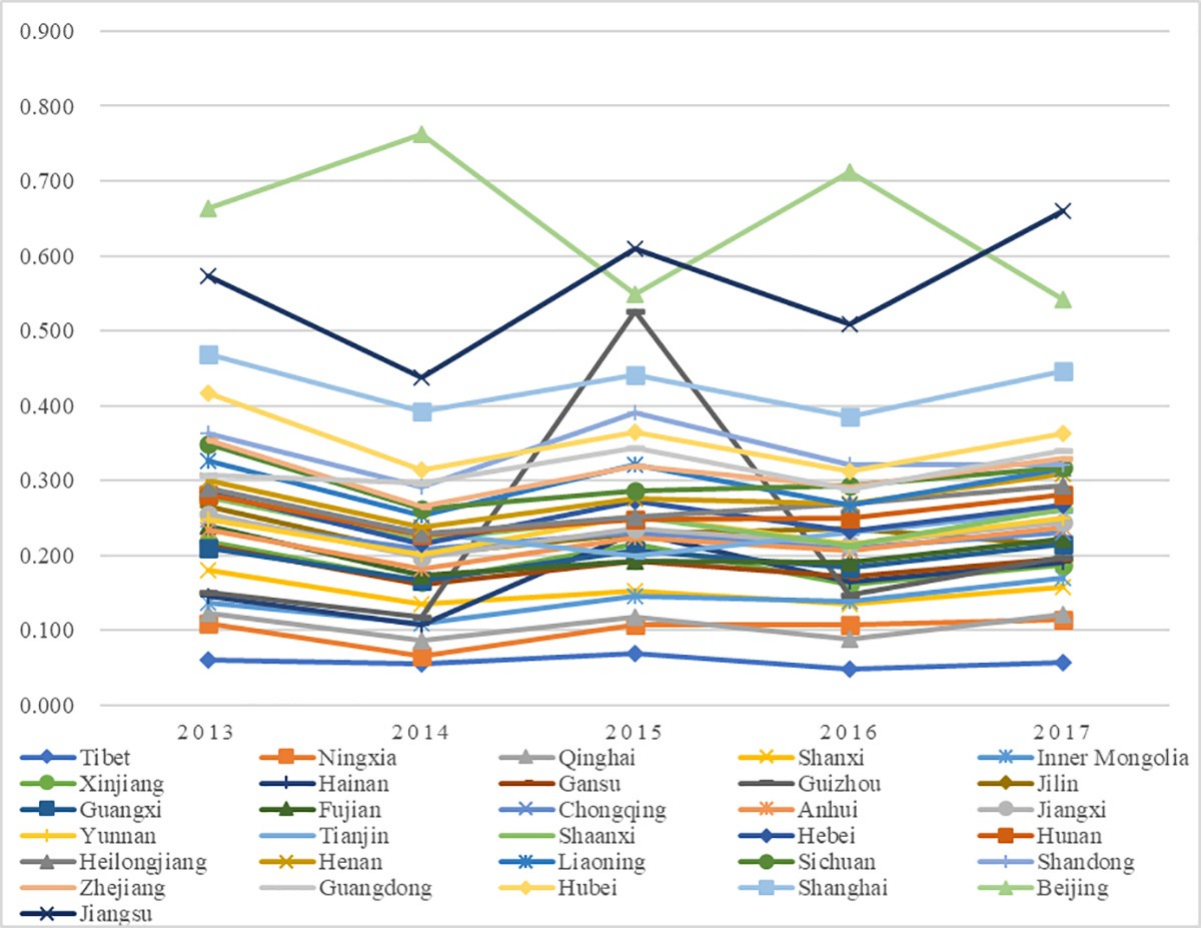
Trends of C-SHED.

The values and ranks of the C-SHED in China from 2013 to 2017 are shown in S1 Table, and the change trends of the values are shown in Fig 3.

Generally, from the temporal perspective, the C-SHEDs in most regions of China from 2013 to 2017 were not high, and the development trends were mildly fluctuant. Only one region (Beijing) reached the grade of “excellent” once (in 2014), and only two regions had values above 0.5 in most years, which was “good” in the evaluation grade of the C-SHED (Beijing and Jiangsu), while most regions had scores below 0.5 and received grades of “fair” or even “poor”. In detail, Beijing and Jiangsu stood at the top of the ranking, with most scores over 0.50 and grades of “good”, proving that these two regions had developed the best C-SHED in China, though their development grades could still be promoted to better status. Specifically, Jiangsu ranked at the top, proving the correctness of a folk saying in China: “For education development, all regions should learn from Jiangsu”; Beijing was competitive mainly due to its status as the national capital and the preferential policies received from the central government. Moreover, Ningxia, Qinghai, and Tibet were the last three during these five years. Located in West China, these three regions lacked innate advantages in higher education, so the C-SHEDs were not satisfactory. Specifically, Tibet consistently ranked last from 2013 to 2017, so more efforts were needed to enhance its C-SHED. In addition, the other regions shared similar situations: they ranked at the middle level (grade “fair” or “poor”) with mildly fluctuating trends. Among them, Guizhou fluctuated greatly in 2015 but remained stable with a “poor” grade in the rest of the years, which was mainly because of the high values of the total revenue of and taxes paid by incubated enterprises in the NUSP in 2015. The regions with fair and poor performance should pay more attention to SHED and upgrade their status.

**Fig 4:**
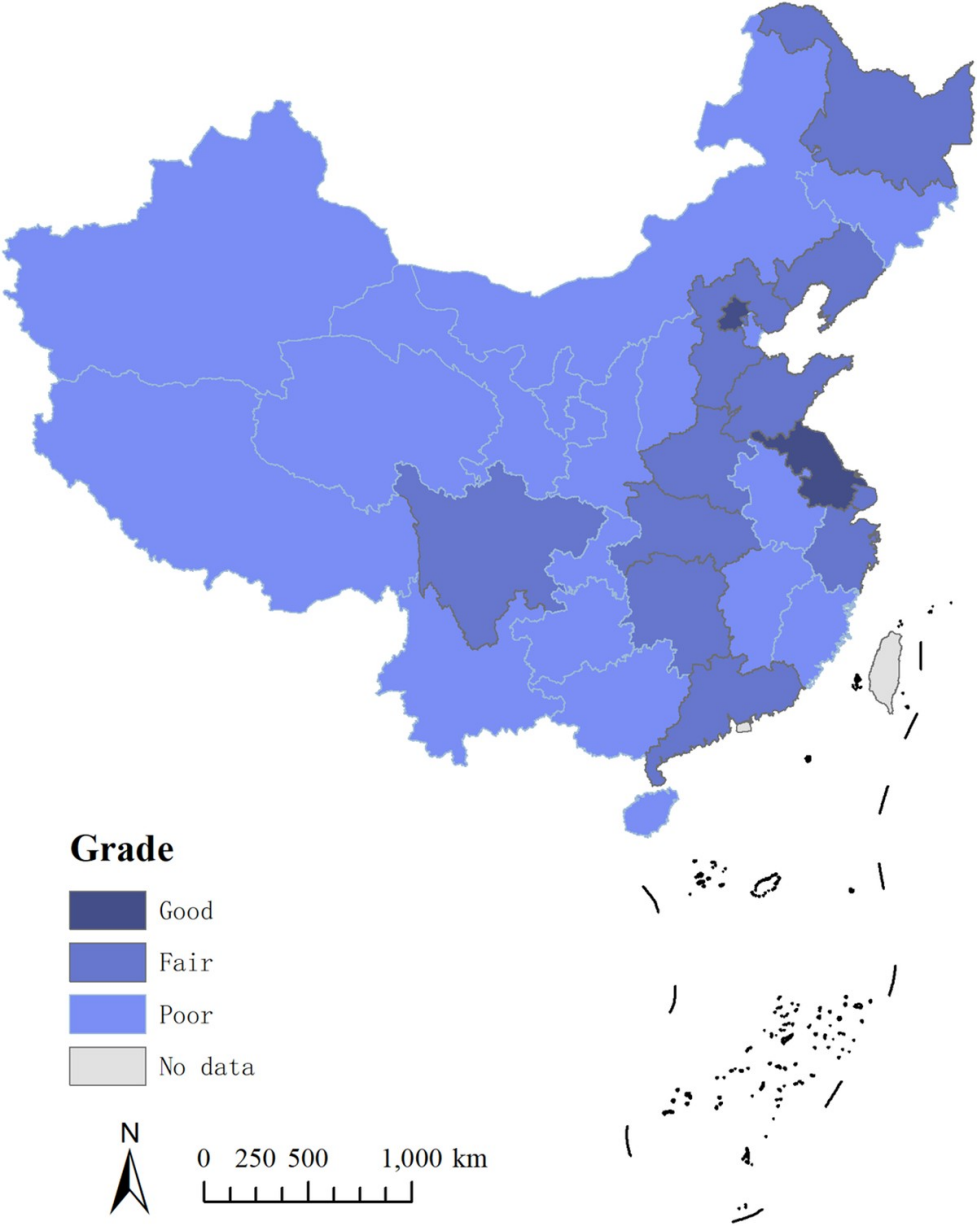
Geographical distribution of the average grade of C-SHED.

The geographical distribution of the average grades of C-SHEDs is visualized by ArcGIS 10.2 (Fig 4); it is shown that there are certain geographical correlations of C-SHEDs in China. From the spatial perspective, the noncoastal regions had relatively lower grades of C-SHEDs, while the coastal regions and the Central-south China regions had higher C-SHEDs, which was mainly due to the geographical, historical and economic advantages of the coastal regions and the Central-south China regions. These regions had a longer history of higher education in China, greater financial support, and key and prestigious universities to lead the local SHED, so these regions enjoyed relatively higher C-SHEDs. In contrast, most areas in the west and noncoastal areas had low grades on C-SHEDs mainly because of the fewer resources for SHED, weaker SHED foundation, and lack of prominent universities as the leading models in SHED.

### Analysis of D-SHED

**Fig 5:**
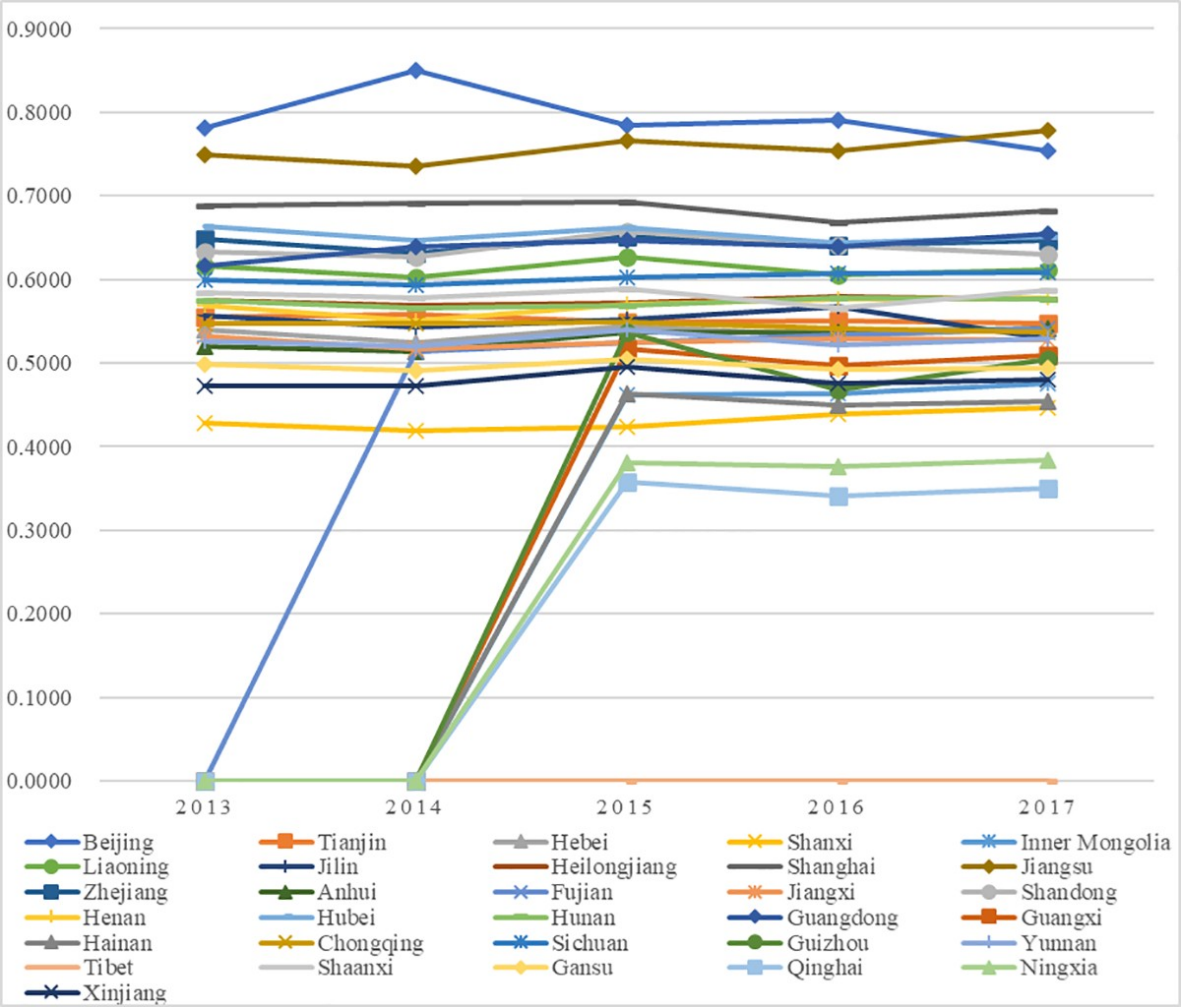
Trends of D-SHED.

The D-SHED values are shown in S2 Table, and the temporal trends of D-SHED are shown in Fig 5.

From the temporal perspective, the coordination degrees of the regional economy, effectiveness, efficiency, equity, earnings and equality of SHED coupling in most regions of China from 2013 to 2017 mainly remained stable in the respective classifications, and a few regions experienced great increases. Among them, Fujian, Inner Mongolia, Guangxi, Hainan, Guizhou, Qinghai, and Ningxia witnessed large changes from high imbalance in 2013, with values of 0, to higher coordination classifications in 2017. Specifically, Fujian changed from high imbalance in 2013 to slight coordination in 2014 (0.5145) and kept that status in the following years; it leaped up 4 levels. Guangxi and Guizhou maintained high imbalance in 2013 and 2014 and then changed to slight coordination in the remaining years (0.5085 and 0.5043, respectively, in 2017), indicating that the D-SHEDs of these regions maintained relatively moderate levels in the remaining years. Inner Mongolia and Hainan maintained high imbalance in 2013 and 2014 and then approached imbalance in the next 3 years (0.4758 and 0.4542, respectively, in 2017); Qinghai and Ningxia presented a slight imbalance after 2015 (0.3578 and 0.3813), illustrating that these regions experienced transition periods from imbalanced to average in recent years. These regions changed greatly mainly because of their low D-SHED status at the beginning (0 in 2013), which was caused by the imbalanced coordination in the economy dimension: there were no NUSPs in these regions, so no values were available in this dimension. Compared with the other 5 dimensions, the economy dimension presented highly imbalanced coordination development, which led to a high imbalance of the D-SHED for these regions.

On the other hand, the remaining regions maintained a relatively stable status. Among them, Tibet was a special case, as its D-SHED maintained the value 0, meaning that it maintained the status of high imbalance for the entire 5-year period. This was because there were no NUSPs in Tibet, which led to an imbalance between the economic dimension and the other dimensions that has persisted until the present. In addition, Beijing and Jiangsu maintained high D-SHEDs (above 0.7), and Beijing even enjoyed the status of high coordination in 2014 (0.8495), demonstrating that the two regions maintained coordination among the 6 dimensions in SHED. Furthermore, other regions held relatively stable status, and the D-SHEDs fluctuated between the level of approaching imbalance (0.4–0.5) and primary coordination (0.6–0.7), proving that the SHEDs of these regions have remained coordinated to some degree in recent years. However, it is also noticeable that the trend of Beijing was declining slightly (from 0.7811 in 2013 to 0.7534 in 2017), so a comprehensive approach should be taken to optimize the D-SHED and to prevent potential degeneration.

**Fig 6:**
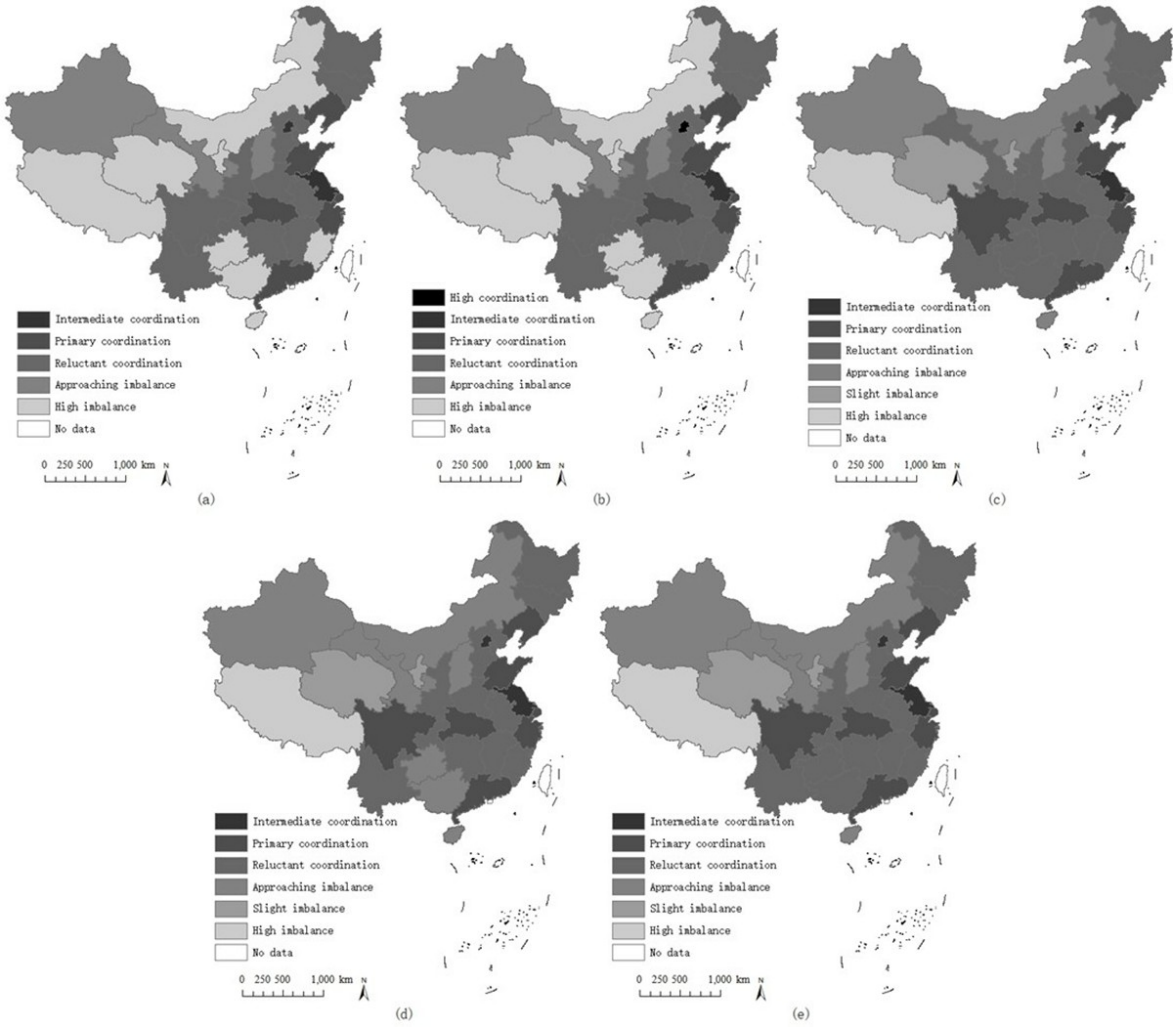
Geographical distribution of D-SHEDs. (a) 2013; (b) 2014; (c) 2015; (d) 2016; (e) 2017.

To analyze the spatial changes of the D-SHEDs dynamically, ArcGIS 10.2 was utilized to visualize the results from 2013 to 2017, as shown in Fig 6. There are two findings from the figure. (1) The D-SHED in China was generally improving over the five years because increasing numbers of regions were achieving higher coupling coordination classifications, especially Guizhou, Guangxi, Fujian, Inner Mongolia, Ningxia, Qinghai, and Hunan: the D-SHEDs of these regions obviously increased from lower classifications to higher ones. The general improvement of the D-SHEDs demonstrated that the dimensions of sustainable higher education developed coordinatively and harmoniously, and related policies aiming to enhance and promote the sustainable higher education development and competitiveness proposed by the authorities had positive effects. (2) The D-SHEDs in the western regions were lower than those in the central and eastern regions of China; however, the gap between the west and other regions was declining. This is consistent with the current reality in China: although the western regions of China were less developed in terms of SHED due to historical and geographical reasons, the gap among regions was diminishing because of the special policies or strategies for western regions, such as the West China Development Strategy and the Special Foundations for Western University Policy, so the western regions were gradually catching up with other places. However, although the gap is declining, regional differences in D-SHEDs still exist; therefore, corresponding countermeasures are required for different regions to effectively and efficiently enhance D-SHEDs.

## Countermeasures

The relatively stable statuses of SHED and the geographical differences among regions indicate that it is necessary to take corresponding and specific countermeasures to accelerate higher education development with the philosophy of sustainability in different regions. Therefore, the following countermeasures are proposed for further effective and efficient actions.

For the western regions, where C-SHEDs and D-SHEDs are relatively low, great efforts are needed to enhance SHED in almost every aspect. Potential countermeasures are as follows.

(1) Compensate for innate weaknesses (such as developing national university science parks) and make use of intrinsic advantages (such as accentuating the leading role of the local key universities) to achieve benign coordination among the dimensions of higher education and realize the sustainable development of higher education. For instance, it is encouraged that Tibet compensate for the shortage in SHED by setting up national university science parks. In addition, it is possible for Gansu to explore the innate potential of its local key universities and support those institutions as the engines of SHED.

(2) Make use of supportive policies (such as the policy of West China Development and the Special Foundations for Western University Policy) to construct mechanisms for interaction with the high SHED regions. In detail, western regions can cooperate with advanced regions in the east and learn both the philosophy and practical skills related to SHED from them. For instance, Ningxia in the west can initiate co-research programs with eastern regions so that it can enhance the research outputs in the effectiveness dimension.

(3) Make use of the geographical advantages of border regions to achieve international cooperation (such as SHED international forums or co-research programs) so that leapfrog development in sustainable higher education can be realized. For instance, Tibet, Xinjiang and Inner Mongolia can initiate international cooperation with neighboring countries, such as India, Russia and Mongolia, under the national strategic frameworks to enhance higher education equality.

For most central regions, with a SHED status at the intermediate level, the focus should be on individual characteristics and targeted actions to enhance SHED. Several actions are proposed, as follows.

(1) Make more precise positioning of the local SHED (e.g., higher education aiming to serve the local economic and social development, support national strategies, lead global changes) and develop sustainable higher education with a differentiation strategy. Analyzing the characteristics carefully and taking targeted actions are necessary; for example, it is necessary for Henan to reconsider its SHED strategy, reposition the NUSP and enhance the contribution of the NUSP by attracting more synergistic industries so that the coordination status of SHED can be improved in the economy dimension.

(2) Establish cross-regional or cross-disciplinary higher education alliances, utilize outside resources and platforms, form resource-sharing and cooperation mechanisms, and create win-win situations. For example, it is possible for Heilongjiang to encourage the participation of private capital in SHED, construct integrated platforms where cooperation between universities and corporations is possible, and enhance the coupling coordination status of SHED in the equity dimension.

For most coastal regions, where SHED and the coupling coordination degrees are high, on the premise of keeping the status quo, it is necessary to optimize the structure of SHED and enhance the overall performances. Detailed countermeasures could be taken, as follows.

(1) Encourage innovation to find new growth points of SHED by introducing supportive policies, such as special supportive policies for innovation programs facilitating SHED and tax-reducing policies for enterprises engaged in technological innovation in the higher education field. For example, in the context of constructing the Shanghai Free Trade Zone, Shanghai can propose new policies to support HEIs and related corporations to initiate innovation programs and accelerate the sustainable development of higher education.

(2) Set up special funds to encourage multilateral participation in exploring the new modes and the future trends of SHED and therefore guarantee the competitiveness of SHED. For example, as the top 2 regions, Beijing and Jiangsu can spend more of their budgets on supporting researchers to explore new modes of SHED and to share their advanced experiences with other regions. In detail, Beijing needs to devote more funds to reverse its downward trend of SHED.

## Conclusions and prospects

This paper selects the 31 provincial regions of China as cases, studies the comprehensive status and the coupling coordination level of sustainable higher education development with IEW-TOPSIS and the coupling coordination model, and makes the following conclusions.

(1) At present, the C-SHEDs in most regions of China are not high, and the development trends are mildly fluctuating. Spatially, there are geographical correlations of C-SHEDs in China: the coastal regions and the Central-south China regions have higher grades of C-SHEDs, and the noncoastal regions have relatively lower C-SHEDs.

(2) Currently, the D-SHEDs in most regions of China mainly remain stable in their respective classifications, and a few regions have experienced great increases. Over the five-year period studied, the D-SHED in China is generally improving; in addition, the D-SHEDs in the western regions are lower than those in the middle and eastern regions; however, the gap between the west and the east is declining.

(3) The philosophy of sustainability is an effective and necessary tool to enhance the SHED in China, and there is still a long way to go. Corresponding specific countermeasures are required for different regions to achieve coordinated SHED development.

There are several contributions of this paper, which are as follows.

(1) This paper constructs the 6E evaluation framework, which is a new and advanced framework compared with the previous frameworks used to measure SHED; based on this framework, this paper then constructs the evaluation index system with indicators that are applicable to objectively and comprehensively evaluate SHED. The framework and the index system well analyze the development and coordination status of sustainable higher education.

(2) This paper uses IEW-TOPSIS and the coupling coordination model to measure the comprehensive development status and the coupling coordination level of SHED, and it proves that IEW-TOPSIS and the coupling coordination model are effective in measuring the performance of higher education. In addition, this paper addresses the deficiencies of former studies, which mainly focus on a single case and analyze the coupling relationship between only 2 dimensions.

(3) According to the unique characteristics of the regions, this paper proposes specific and practical countermeasures that are suitable for different places. These countermeasures contribute to a better understanding of the coupling coordination relationships of SHED in 6 dimensions and contribute to better application of the 6E evaluation framework in SHED practice.

There are some limitations. The evaluation index system should be multidimensional with representative indicators, but due to the limited accessibility of certain data, some relevant indicators or factors must be ignored (e.g., international cooperation); furthermore, due to their significant differences, certain regions, such as Hong Kong, Macau and Taiwan, are also neglected, which makes this study not perfect. These limitations are prospects for future research and merit further discussion.

## Supporting information

S1 Table Value and rank of C-SHED. (PDF)

S2 Table Value of D-SHED. (PDF)
