# Supplementary material for: Measurement of sustainable higher education development: Evidence from China
Source: PLoS One. 2020 Jun 1;15(6):e0233747. doi: 10.1371/journal.pone.0233747 (PMC7263635; doi:10.1371/journal.pone.0233747)
Supplement: S1 Table — (PDF) [file pone.0233747.s001.pdf]

S1 Table. Value and Rank of C-SHED.

	2013		2014		2015		2016		2017	
	**Value**	**Rank**	**Value**	**Rank**	**Value**	**Rank**	**Value**	**Rank**	**Value**	**Rank**
Beijing	0.663	1	0.763	1	0.549	2	0.712	1	0.541	2
Tianjin	0.279	14	0.231	11	0.199	24	0.231	15	0.260	15
Hebei	0.285	12	0.216	15	0.272	12	0.232	14	0.266	13
Shanxi	0.180	25	0.134	25	0.152	27	0.135	28	0.158	28
Inner Mongolia	0.137	28	0.108	27	0.146	28	0.139	27	0.169	27
Liaoning	0.325	8	0.254	9	0.321	8	0.267	11	0.316	9
Jilin	0.266	16	0.201	17	0.228	20	0.235	13	0.214	22
Heilongjiang	0.290	11	0.229	12	0.251	15	0.269	9	0.294	11
Shanghai	0.469	3	0.393	3	0.442	4	0.385	3	0.446	3
Jiangsu	0.574	2	0.437	2	0.610	1	0.509	2	0.660	1
Zhejiang	0.355	6	0.266	7	0.319	9	0.291	7	0.330	6
Anhui	0.234	21	0.182	20	0.224	21	0.207	20	0.237	18
Fujian	0.239	20	0.173	21	0.192	26	0.191	21	0.222	20
Jiangxi	0.255	17	0.198	19	0.235	17	0.215	17	0.245	17
Shandong	0.362	5	0.292	6	0.391	5	0.321	4	0.321	7
Henan	0.299	10	0.237	10	0.276	11	0.269	10	0.308	10
Hubei	0.416	4	0.314	4	0.365	6	0.313	5	0.362	4
Hunan	0.280	13	0.227	13	0.249	16	0.249	12	0.280	12
Guangdong	0.308	9	0.296	5	0.343	7	0.288	8	0.341	5
Guangxi	0.209	24	0.167	22	0.206	23	0.183	22	0.214	21
Hainan	0.145	27	0.107	28	0.231	19	0.164	24	0.191	25
Chongqing	0.249	18	0.209	16	0.231	18	0.210	19	0.231	19
Sichuan	0.348	7	0.261	8	0.287	10	0.293	6	0.317	8
Guizhou	0.151	26	0.117	26	0.525	3	0.147	26	0.198	23

Yunnan	0.248	19	0.201	18	0.253	13	0.215	16	0.250	16
Tibet	0.061	31	0.055	31	0.069	31	0.048	31	0.058	31
Shaanxi	0.278	15	0.221	14	0.251	14	0.212	18	0.262	14
Gansu	0.212	23	0.161	24	0.193	25	0.172	23	0.195	24
Qinghai	0.123	29	0.087	29	0.117	29	0.088	30	0.121	29
Ningxia	0.109	30	0.065	30	0.108	30	0.108	29	0.115	30
Xinjiang	0.218	22	0.164	23	0.214	22	0.162	25	0.187	26
