# Supplementary material for: Measurement of sustainable higher education development: Evidence from China
Source: PLoS One. 2020 Jun 1;15(6):e0233747. doi: 10.1371/journal.pone.0233747 (PMC7263635; doi:10.1371/journal.pone.0233747)
Supplement: S2 Table — (PDF) [file pone.0233747.s002.pdf]

S2 Table. Value of D-SHED.

Region	2013	2014	2015	2016	2017
Beijing	0.7811	0.8495	0.7846	0.7910	0.7534
Tianjin	0.5548	0.5582	0.5498	0.5502	0.5469
Hebei	0.5401	0.5253	0.5441	0.5316	0.5416
Shanxi	0.4288	0.4184	0.4232	0.4395	0.4471
Inner Mongolia	0.0000	0.0000	0.4622	0.4635	0.4758
Liaoning	0.6162	0.6028	0.6265	0.6059	0.6123
Jilin	0.5561	0.5429	0.5523	0.5676	0.5297
Heilongjiang	0.5744	0.5694	0.5713	0.5789	0.5771
Shanghai	0.6878	0.6907	0.6934	0.6685	0.6818
Jiangsu	0.7489	0.7361	0.7654	0.7545	0.7782
Zhejiang	0.6480	0.6309	0.6509	0.6409	0.6475
Anhui	0.5206	0.5134	0.5368	0.5367	0.5419
Fujian	0.0000	0.5145	0.5251	0.5335	0.5418
Jiangxi	0.5326	0.5166	0.5242	0.5285	0.5274
Shandong	0.6328	0.6271	0.6580	0.6402	0.6294
Henan	0.5684	0.5513	0.5706	0.5761	0.5781
Hubei	0.6636	0.6465	0.6626	0.6445	0.6519
Hunan	0.5743	0.5666	0.5694	0.5774	0.5766
Guangdong	0.6168	0.6399	0.6468	0.6386	0.6548
Guangxi	0.0000	0.0000	0.5166	0.4976	0.5085
Hainan	0.0000	0.0000	0.4627	0.4502	0.4542
Chongqing	0.5480	0.5473	0.5498	0.5407	0.5368
Sichuan	0.5992	0.5932	0.6019	0.6077	0.6087
Guizhou	0.0000	0.0000	0.5364	0.4673	0.5043
Yunnan	0.5258	0.5197	0.5397	0.5215	0.5288
Tibet	0.0000	0.0000	0.0000	0.0000	0.0000

Shaanxi	0.5849	0.5777	0.5887	0.5655	0.5876
Gansu	0.4981	0.4915	0.5054	0.4931	0.4939
Qinghai	0.0000	0.0000	0.3578	0.3411	0.3502
Ningxia	0.0000	0.0000	0.3813	0.3769	0.3843
Xinjiang	0.4723	0.4719	0.4950	0.4763	0.4802

2
